# SPECT Imaging Agents for Detecting Cerebral *β*-Amyloid Plaques

**DOI:** 10.1155/2011/543267

**Published:** 2011-04-13

**Authors:** Masahiro Ono, Hideo Saji

**Affiliations:** Graduate School of Pharmaceutical Sciences, Kyoto University, 46-29 Yoshida Shimoadachi-cho, Sakyo-ku, Kyoto 606-8501, Japan

## Abstract

The development of radiotracers for use *in vivo* to image *β*-amyloid (A*β*) plaques in cases of Alzheimer's disease (AD) is an important, active area of research. The presence of A*β* aggregates in the brain is generally accepted as a hallmark of AD. Since the only definitive diagnosis of AD is by postmortem staining of affected brain tissue, the development of techniques which enable one to image A*β* plaques *in vivo* has been strongly desired. Furthermore, the quantitative evaluation of A*β* plaques in the brain could facilitate evaluation of the efficacy of antiamyloid therapies currently under development. This paper reviews the current situation in the development of agents for SPECT-based imaging of A*β* plaques in Alzheimer's brains.

## 1. Introduction

Alzheimer's disease (AD) is an age-related, irreversible form of dementia characterized by memory loss, a progressive decline in intellectual ability, language impairment, and personality and behavioral changes that eventually interfere with daily life. The accumulation of *β*-amyloid (A*β*) aggregates (major protein aggregates of senile plaques) in the brain is considered one of the hallmarks of AD [1, 2]. Today, the clinical diagnosis of AD is primarily based on history and memory testing, which is often difficult and not accurate, as the early cognitive and behavioral symptoms of AD are difficult to distinguish from normal signs of aging. To facilitate the early diagnosis of this disease, there is an urgent need for the sensitive noninvasive detection of biomarkers for the pathophysiology. Toward achieving this goal, nuclear imaging techniques such as positron emission computed tomography (PET) and single photon emission computed tomography (SPECT) have been employed. Radionuclide-labeled agents targeting the A*β* plaques in the brain may greatly facilitate the diagnosis of AD and new antiamyloid therapies [3–7]. The differential diagnosis for AD includes a large number of other diseases such as vascular dementia, frontal temporal lobe dementia (FTLD) complex, and dementia with Lewy bodies (DLB) as well as rarer neurodegenerative diseases such as Creutzfeld-Jacob disease (CJD). Importantly, AD subjects will always have A*β* plaques, whereas A*β* is seen not at all or only sporadically in most of these other diseases. In each case, appropriate prognosis and treatment require accurate diagnostic assessment.

**Figure 1:**
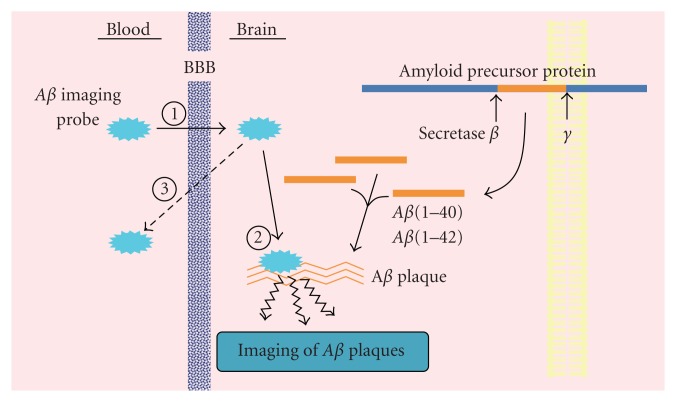
Strategy of* in vivo* imaging of cerebral A*β* plaques.

**Figure 2:**
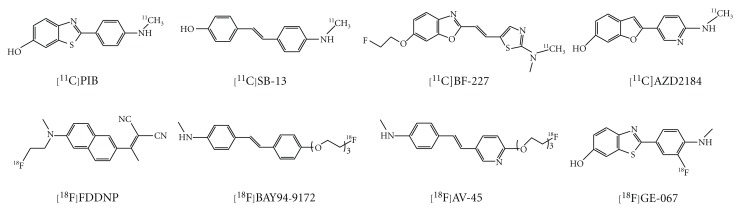
Chemical structure of PET imaging agents tested clinically.

Developing A*β* imaging agents is currently an emerging field of research. The basic requirements for suitable A*β* imaging agents include (i) good penetration of the blood-brain barrier, (ii) selective binding to A*β* plaques, and (iii) clear and contrasting signals between plaques and nonplaques (Figure 1). Based on these requirements, several promising agents with the backbone structure of DDNP, thioflavin-T and Congo Red have been synthesized and evaluated for use *in vivo* as probes to image A*β* plaques in AD brain. Clinical trials in AD patients have been conducted with several PET imaging agents including [^11^C]PIB [8–10], [^11^C]SB-13 [6, 11], [^11^C]BF-227 [12], [^11^C]AZD2184 [13], [^18^F]FDDNP [14–16], [^18^F]BAY94-9172 [7, 17, 18], [^18^F] AV-45 [19–21], and [^18^F]GE-067 [22] (Figure 2), indicating the imaging of A*β* plaques in living brain tissue to be useful for the diagnosis of AD. The ^11^C-labeled agents limit their use to on-site cyclotrons and sophisticated radiochemistry laboratories due to the short half-life (20 min) of ^11^C. PET agents with the longer half-life (110 min) radioisotope ^18^F have recently been developed and could increase the availability of A*β* imaging to all PET facilities, but still represents a minority of modern hospitals, as only a small fraction of hospitals have a PET scanner. Since SPECT is more valuable than PET in terms of routine diagnostic use, the development of more useful A*β* imaging agents for SPECT has been a critical issue. However, progress in developing imaging agents targeting A*β* plaques is less advanced for SPECT than PET. In this review, we summarize the current situation in the development of probes for SPECT-based imaging of A*β* plaques in Alzheimer's brains.

## 2. Radioiodinated Probes for Imaging of A*β* Plaques

**Figure 3:**
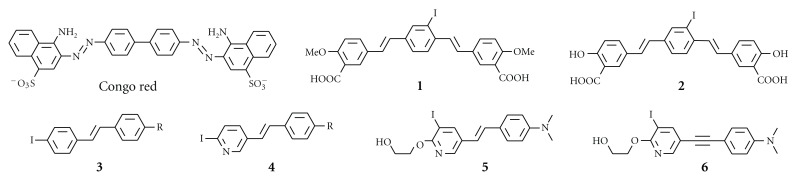
Chemical structure of SPECT imaging agents derived from Congo Red.

**Figure 4:**
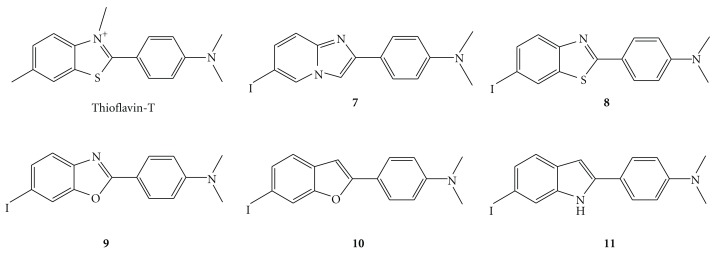
Chemical structure of SPECT imaging agents derived from thioflavin T.

Many radioiodinated imaging agents derived from Congo Red or thioflavin-T have been developed. Compounds **1 **[29], **2 **[29], **3 **[40], **4 **[23], **5 **[24], and **6 **[25] (Figure 3) are thought to be derived from Congo Red. Although **1**, **2**, and **3** showed unfavorable pharmacokinetics *in vivo* such as low uptake into the brain and a slow washout, the radioactivity pharmacokinetics of** 5** and **6** was much improved. Because thioflavin-T has a lower molecular weight than Congo Red, implying greater blood-brain penetration, a number of groups have worked to develop probes for SPECT derived from thioflavin-T including **7 **(IMPY) [26–28], **8** (TZDM) [29], **9** (IBOX) [30], **10** (benzofuran derivatives) [31], and **11** (phenylindole derivatives) (Figure 4) [32]. 

Initially, Zhuang and coworkers prepared iodo-strylbenzene derivatives based on the chemical structure of Congo Red, [^125^I]IMSB (**1**) and [^125^I]ISB (**2**). These ligands exhibited low brain uptake likely due to two ionizable carboxyl groups [29]. Thus, a small and neutral thioflavin-T analog, [^125^I]TZDM (**8**), was prepared [29]. *In vitro* binding studies of these ligands, [^125^I]ISB, [^125^I]IMSB and [^125^I]TZDM, showed excellent binding affinities with *K*_*d*_ values of 0.08, 0.13 and 0.06 nM for aggregates of A*β*(1–40) and 0.15, 0.73 and 0.14 nM for aggregates of A*β*(1–42), respectively. Interestingly, in a competitive-binding assay, different binding sites on A*β*(1–40) and A*β*(1–42) aggregates, which are mutually exclusive, were observed for Congo Red and thioflavin-T derivatives. Biodistribution experiments in normal mice after an i.v. injection showed that [^125^I]TZDM exhibited good uptake and retention in the brain, much higher than [^125^I]ISB and [^125^I]IMSB. Preliminary experiments on the biodistribution of [^125^I]TZDM in transgenic mice, engineered to produce excess A*β* plaques in the brain as an AD model, suggested labeling of A*β* aggregates *in vivo*. However, [^125^I]TZDM is not ideal as an imaging agent *in vivo*, due to its labeling of white matter, which significantly increases the background activity. To improve the pharmacokinetics of uptake and retention, Kung et al. and others have prepared several compounds derived from thioflavin-T and studied features such as affinity for A*β* aggregates* in vitro* and biodistribution* in vivo*. Interestingly, the compounds with good binding to A*β* aggregates share a common structural feature: either an *N*-methylamino- or *N*,*N*-dimethylaminophenyl group at one end of the molecule. The structural feature required for binding to A*β* aggregates appears to be simple and unique.

 [^123^I]IMPY has been characterized as a potential agent for SPECT-based imaging of A*β* plaques. IMPY displayed selective labeling of A*β* plaques *ex vivo* in autoradiographic experiments using double-transgenic mice (PSAPP) as a model of AD [33]. Preliminary clinical data on [^125^I]IMPY in normal and AD patients showed a distinct distribution pattern similar to that of [^11^C]PIB [34, 35]. However, the signal-to-noise ratio for plaque labeling is not as high as that of [^11^C]PIB. The low contrast may be due to the fast clearance from brain and plasma observed in AD and normal subjects. But the rapid metabolism and instability of [^123^I]IMPY *in vivo* may have led to less than optimal signal-to-noise ratios for targeting A*β* plaques in the brain. Additional candidates are being explored for SPECT imaging of A*β* plaques in the brain.

**Figure 5:**
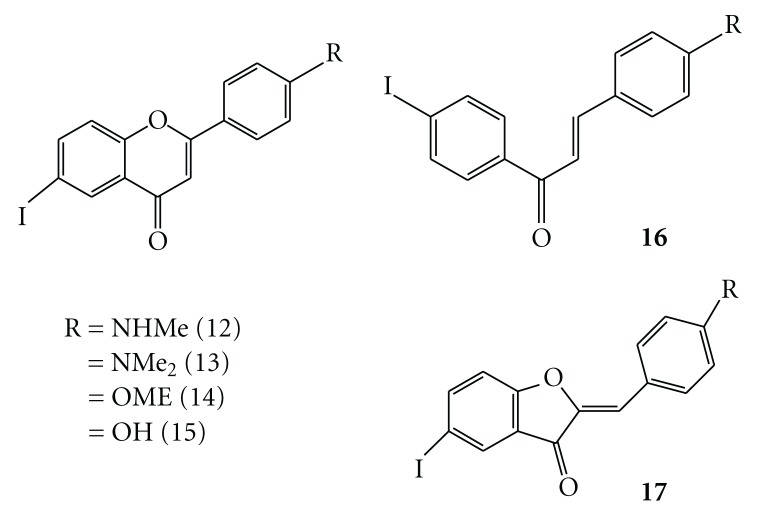
Chemical structure of SPECT imaging agents based on flavone (**12**–**15**), chalcone (**16**), and aurone (**17**).

Recently, the effects of polyhydroxyflavones on the formation, extension, and destabilization of A*β* aggregates have been studied *in vitro *[36]. These flavones dose-dependently inhibited the formation of A*β* aggregates, as well as destabilized preformed A*β* aggregates, indicating that they could interact directly with the aggregates. The findings in that report prompted us to use flavones as a core structure in the development of A*β* imaging agents. Furthermore, some recent studies have shown that electron-donating groups such as methylamino, dimethylamino, methoxy, and hydroxy groups play a critical role in the binding to A*β* aggregates. With these considerations in mind, we designed four radioiodinated flavones with a radioiodine at the 6-position and an electron-donating group at the 4′-position (Figure 5). Then we synthesized a series of flavone derivatives and evaluated their usefulness *in vivo *as SPECT A*β* imaging agents [37]. 

**Figure 6:**
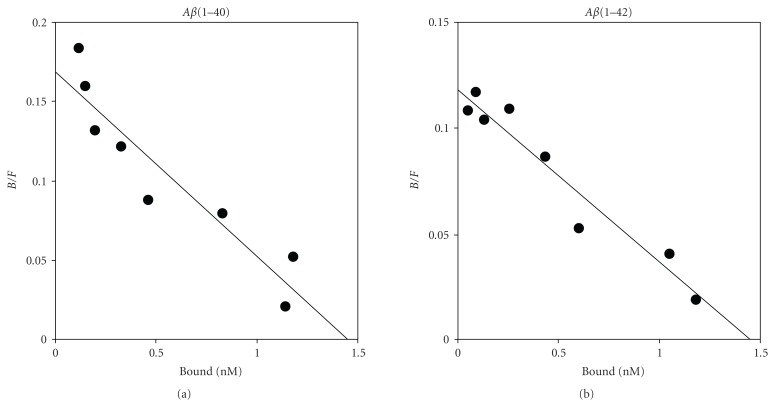
Scatchard plots of the binding of [^125^I]**12** to aggregates of A*β*(1–40) (a) and A*β*(1–42) (b).

Experiments on the binding of [^125^I]**12** to aggregates of A*β*(1–40) and A*β*(1–42) were carried out. Transformation of the saturation binding of [^125^I]**12** to Scatchard plots gave linear plots, suggesting one binding site (Figure 6). [^125^I]**12** showed excellent affinity for both A*β*(1–40) (*K*_*d*_ = 12.4 ± 2.3 nM) and A*β*(1–42) (*K*_*d*_ = 17.4 ± 5.7 nM) aggregates. The binding of nonradioactive flavone derivatives (compounds **12**, **13**, **14**, and **15**) was evaluated in experiments inhibiting [^125^I]**12** from binding A*β*(1–40) and A*β*(1–42) aggregates. As shown in Table 1, all flavone derivatives competed well with [^125^I]**12** (*K*_*i*_ = 13–77 nM). More interestingly, when thioflavin-T, and Congo Red gave high *K*_*i*_ values (>1000 nM) (Table 1), indicating little competition. This finding suggests that these flavones may have a binding site on A*β* aggregates different from that of thioflavin T and Congo Red, although additional studies regarding the selectivity of binding affinity for A*β* aggregates are required.

**Figure 7:**
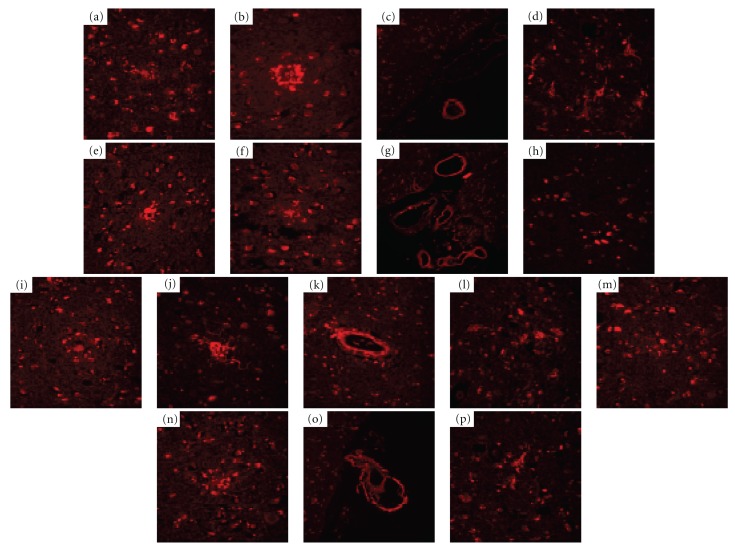
Neuropathological staining of compounds **12 **(a)–(d), **13** (e)–(h), **14 **(i)–(l), and **15 **(m)–(p) on 5 *μ*m AD brain sections from the temporal cortex. (a) A*β* plaques (a), (e), (i), and (m) are clearly stained with **12**, **13**, **14, **and **15** (×40 magnification). Clear staining of neuritic plaques (b), (f), (j), and (n) and cerebrovascular amyloid (c), (g), (k), and (o) was also obtained. Many NFTs (d), (h), (l), and (p) are intensely stained with **12, 13**, **14**, and **15** (×40 magnification).

Since the *in vitro* binding assays demonstrated the high affinity of the flavone derivatives for A*β*(1–40) and A*β*(1–42) aggregates, compounds** 12, 13**,** 14**, and** 15** were investigated for their neuropathologic staining of A*β* plaques and NFTs in human AD brain sections (Figure 7). The compounds intensely stained A*β* plaques (Figures 7(a), 7(e), 7(i), and 7(m)), neuritic plaques (Figures 7(b), 7(f), 7(j), and 7(n)), and cerebrovascular amyloids (Figures 7(c), 7(g), 7(k), and 7(o)) with nearly the same pattern. However, as seen in Figures 7(a), 7(e), 7(i), and 7(m), these flavone compounds did not intensely stain the core region in so-called classic A*β* plaques, unlike the thioflavin-T and Congo Red derivatives previously reported as A*β* imaging probes, indicating that flavone derivatives may have somewhat distinct binding characteristics for amyloid fibrils. These flavone derivatives appear to stain not only neuritic A*β* plaques but also diffuse amyloid plaque deposits, which are known to be mainly composed of A*β*(1−42) [38] and to be the initial pathologic change in AD [39]. Thus flavone derivatives with high affinity for A*β*(1–42)-positive diffuse plaques may be more useful for presymptomatic detection of AD. Furthermore, **12, 13**,** 14**, and** 15** also showed high affinity for NFTs in AD brain sections (Figures 7(d), 7(h), 7(l), and 7(p)). These findings suggest that these flavone derivatives can bind amyloid fibrils and NFTs without the backbone structure of thioflavin-T or Congo Red and that quantitative evaluation of their cerebral localization may provide useful information on A*β* and tau pathology.

Four radioiodinated flavone ligands ([^125^I]**12**, [^125^I]**13**, [^125^I]**14,** and [^125^I]**15**) were evaluated for their biodistribution* in vivo* in normal mice. Previous studies suggest that the optimal lipophilicity range for brain entry is observed for compounds with log *P-*values between 1 and 3 [5]. All four ligands displayed optimal lipophilicity as reflected by log *P-*values of 1.94, 2.69, 2.41, and 1.92, respectively. As expected, these ligands exhibited high uptake ranging from 3.2% to 4.1% ID/g brain at 2 min postinjection, a level sufficient for imaging in the brain (Table 2). In addition, they displayed good clearance from the normal brain: 1.2, 1.0, 0.17, and 0.08% ID/g at 60 min postinjection for [^125^I]**12**, [^125^I]**13**, [^125^I]**14**, and [^125^I]**15**, respectively. Radioiodinated amyloid imaging agents such as [^125^I]*m*-I-stilbene (**3**) [40], [^125^I]TZDM (**8**) [29], [^125^I]IBOX (**9**) [30], and [^125^I]benzofuran (**10**) [31], and [^125^I]phenylindole (**11**) [32] reported previously showed good uptake, but a relatively slow washout from the normal brain. A low washout rate leads to high background activity and prevents the visualization of A*β* plaques in the AD brain. Appropriate properties* in vivo* (higher uptake and faster washout from the normal brain) make radioiodinated flavones useful candidates for SPECT tracers for A*β* imaging.

On the basis of this success in the development of SPECT imaging agents, to search for more useful candidates for A*β* imaging probes, we have designed a chemical modification of the flavone structure, and selected the chalcone and aurone structure as a novel core for A*β* imaging probes (Figure 5) [41, 42]. Chalcone and aurone are categorized as flavonoids containing a flavone. We newly designed and synthesized novel chalcone and aurone derivatives, and evaluated the effect of their structure–activity relationships on binding to A*β* aggregates and biodistribution *in vivo* using a compound with high affinity [42–45]. Currently, SPECT imaging agents based on chalcone and aurone are optimized. 

**Figure 8:**
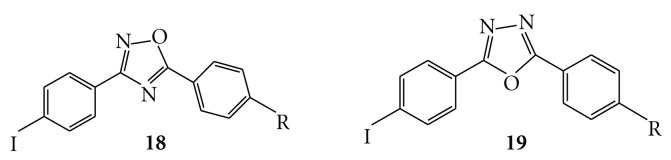
Chemical structure of diphenyl oxadiazoles.

Most of the A*β* imaging probes reported previously have two aromatic rings. Among them, 1,4-diphenyltriazole and 2,5-diphenylthiophene derivatives have triazole and thiophene between two benzene rings, respectively, and it has been shown that they have high-binding affinity for binding to A*β* aggregates despite the kinds of substituted groups [46, 47]. In an attempt to further develop novel ligands for the imaging of A*β* plaques in AD, we designed a series of 3,5-diphenyl-1,2,4-oxadiazole (**18**) [48, 49] and 2,4-diphenyl-1,3,5-oxadiazole (**19**) [49] derivatives (Figure 8). Although the diphenyloxadiazole pharmacophore with high-binding affinity for A*β* aggregates may be useful as a backbone structure to develop novel A*β* imaging agents, additional modifications are necessary to improve the uptake and rapid clearance of nonspecifically bound radiotracers. 

Many factors such as molecular size, ionic charge, and lipophilicity affect the brain uptake of compounds. Since lipophilicity of the compounds generally increases by introduction of iodine, the large higher lipophilicity of the radioiodinated compounds may constitute one reason for the low brain uptake. In the future, introduction of hydrophilic substituted groups into the amyloid-binding scaffolds will be required to develop more promising radioiodinated tracers with in favorable *in vivo* pharmacokinetics. 

## 3. ^99m^Tc Complexes for Imaging of A*β* Plaques

^99m^Tc (*T*_1/2_ = 6.01 h, 141 keV) has become the most commonly used radionuclide in diagnostics for SPECT, because it is readily produced by an  ^99^Mo/^99m^Tc generator, the medium gamma-ray energy it emits is suitable for detection, and its physical half-life is compatible with the biological localization and residence time required for imaging. Its ready availability, essentially 24 h a day, and easiness of use make it the radionuclide of choice. New ^99m^Tc-labeled imaging agents will provide simple, convenient, and widespread SPECT-based methods for detecting and eventually quantifying A*β* plaques in living brain tissue. 

Han and co-workers described a positively charged ^99^Tc-complex of Congo red (**20**) which binds to A*β* aggregates *in vitro *[50]. The basic structure of this complex is the Congo red backbone in which the biphenyl moiety is replaced by a bipyridyl moiety capable of complexing Tc in the presence of* tert*-butylisonitrile as a coligand. Although these Tc complexes showed high affinity for A*β* aggregates in vitro, they have not been tested* in vivo*. Dezutter and co-workers reported a ^99m^Tc-labeled conjugate of Congo Red with a monoamide-monoaminedithiol (MAMA) chelating ligand [51]. However, brain uptake of this ^99m^Tc-labeled Congo Red derivative (**21**) was minimal, probably because of its large size and ionized character at physiological pH. Serdons and co-workers reported the synthesis of a neutral ^99m^Tc-labeled derivative of thioflavin-T (**22**), namely a benzothiazole derivative conjugated with a bisamine-bisthiol (BAT) ligand, and its biological characterization [52]. It was demonstrated that the ^99m^Tc-labeled thioflavin-T derivative binds* in vitro *to A*β* plaques. Despite its high lipophilicity and neutral character, the ^99m^Tc complex did not cross the blood-brain barrier to a sufficient degree and thus is not useful for the detection of AD *in vivo*. Recently, Chen et al. reported that the ^99m^Tc-labeled thioflavin T using MAMA as a chelation ligand (**23**) demonstrated the binding to A*β* aggregates in sections of brain tissue from transgenic mice and AD patients [53]. In addition, **23** can penetrate the blood-brain barrier with high initial brain uptake and moderate washout. These results are encouraging for further exploration of their derivatives as imaging agents for A*β* plaques in the brain. 

**Figure 9:**
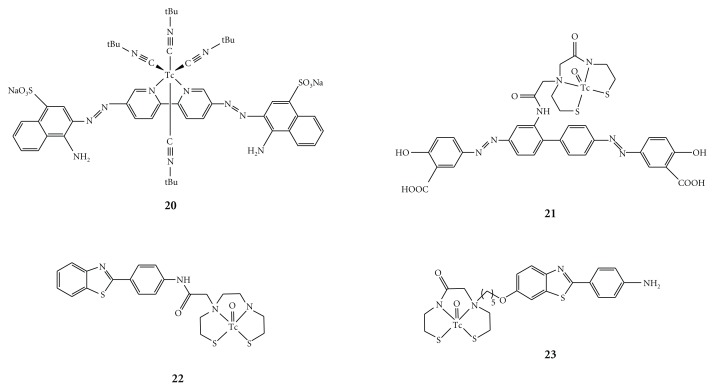
Chemical structure of  ^99m^Tc complexes for imaging of A*β* plaques.

As described above, several ^99m^Tc-labeled imaging probes have been developed (Figure 9) [50–55], but no clinical study of them has been reported. While these ^99m^Tc complexes showed high affinity for A*β* aggregates or A*β* plaques* in vitro*, they suffered the same unfavorable *in vivo* pharmacokinetics in normal mice, that is, a slow washout. Therefore, to make them promising probes for imaging A*β* plaques in the brain, additional molecular modifications to improve their pharmacokinetics *in vivo *are required.

**Figure 10:**
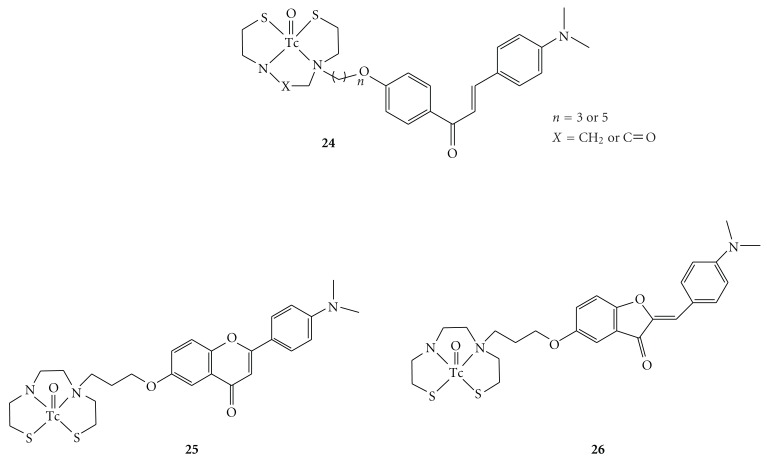
Chemical structure of  ^99 m^Tc complexes based on chalcone (**24**), flavone (**25**), and aurone (**26**) for imaging of A*β* plaques.

**Figure 11:**
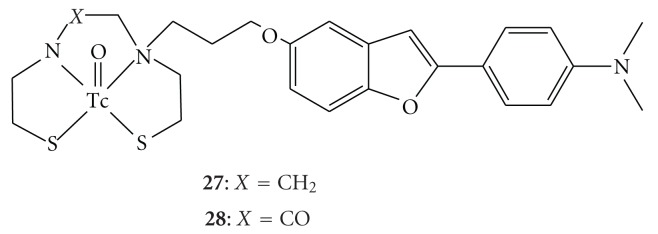
Chemical structure of  ^99m^Tc complexes based on the benzofuran scaffold for imaging of A*β* plaques.

Recently, we have developed several ^99m^Tc complexes based on flavone, chalcone, aurone, and benzofuran derivatives with monoamine-monoamide dithiol (MAMA) and bis-amino-bis-thiol (BAT) as chelation ligands (Figures 10 and 11). MAMA and BAT were selected taking into consideration the permeability of the blood-brain barrier, because they form an electrically neutral complex with ^99m^Tc [56]. We then evaluated their biological potential as probes by testing their affinity for A*β* aggregates and A*β* plaques in sections of brain tissue from Tg2576 mice and their uptake in and clearance from the brain in biodistribution experiments using normal mice. 

Initially, four ^99m^Tc-labeled chalcone derivatives and their corresponding rhenium analogues were tested as potential probes for imaging A*β* plaques (Figure 10) [57]. The chalcones showed higher affinity for A*β*(1–42) aggregates than did ^99m^Tc complexes and, in sections of brain tissue from an animal model of AD, the four Re-chalcones intensely stained A*β* plaques. In biodistribution experiments using normal mice, ^99m^Tc-BAT-chalcone (**24**) displayed high uptake in the brain (1.48%ID/g) at 2 min after injection. The radioactivity washed out from the brain rapidly (0.17%ID/g at 60 min), a highly desirable feature for an imaging agent. Although potential existence of *cis*- and *anti*-isomers was expected, one single isomer was isolated in the preparation of **24**, **25,** and **26**. The chemical identities of **24**, **25,** and **26** were confirmed by NMR and MS, but their absolute configurations have not yet been determined by X-ray crystallography.

**Figure 12:**
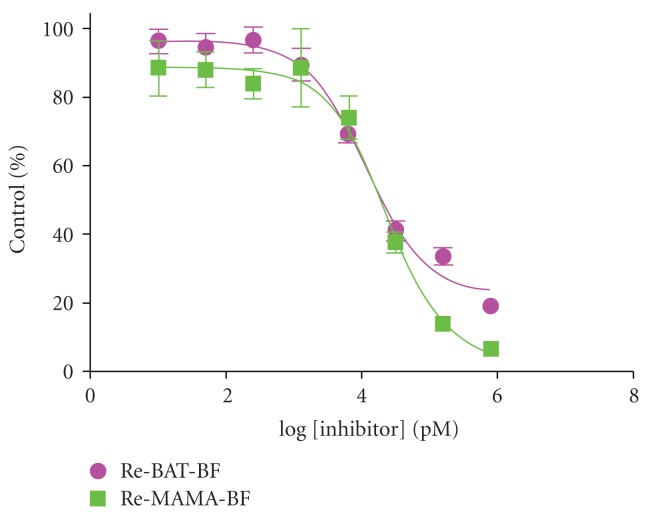
Inhibition curves of Re-BAT-BF and Re-MAMA-BF for the binding of [^125^I]IMPY to A*β*(1–42) aggregates.

**Figure 13:**
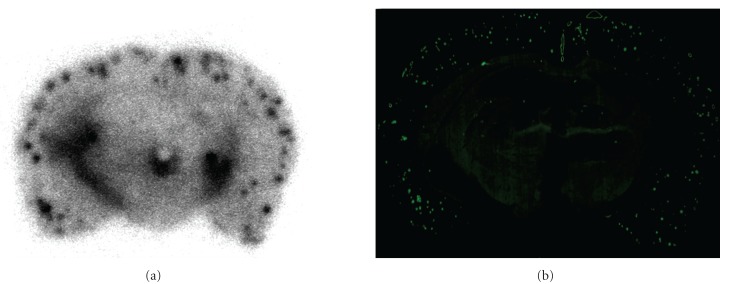
Autoradiography of ^99m^Tc-BAT-BF (**27**) in sections from Tg2576 mouse brain (a). Labeled plaques were confirmed by the staining of the adjacent sections with thioflavin S (b).

As for ^99m^Tc complexes based on benzofuran, we evaluated binding affinity using Re-BAT-BF and Re-MAMA-BF, analogs of ^99m^Tc-BAT-BF (**27**) and ^99m^Tc-MAMA-BF (**28**), respectively. Both ligands inhibited the binding of [^125^I]IMPY to A*β*(1–42) aggregates in a dose-dependent manner, indicating an affinity for A*β* aggregates (Figure 12). Their *K*_*i*_ values were 11.5 and 24.4 nM, respectively, suggesting that Re-BAT-BF displayed higher affinity than Re-MAMA-BF. Next, the affinity of ^99m^Tc-BAT-BF (**27**) for A*β* plaques was investigated *in vitro* using sections of Tg2576 mouse brain (Figure 13). Furthermore, the radioactivity of ^99m^Tc-BAT-BF (**27**) corresponded with the areas of staining with thioflavin S, a dye commonly used for A*β* plaques. In contrast, normal mouse brain displayed no detectable accumulation of  ^99m^Tc-BAT-BF (**27**). The results suggest that ^99m^Tc-BAT-BF (**27**) binds to A*β* plaques in the mouse brain in addition to synthetic A*β* aggregates. 

The biodistribution of ^99m^Tc-BAT-BF (**27**) and ^99m^Tc-MAMA-BF (**28**) was examined in normal mice (Table 3). ^99m^Tc-BAT-BF (**27**) showed greater uptake (1.34%ID/g) than ^99m^Tc-MAMA-BF (**28**) (0.74%ID/g) at 2 min after injection. The uptake of  ^99m^Tc-BAT-BF (**27**) peaked at 10 min after injection, reaching 1.37%ID/g, sufficient uptake for A*β* imaging, and 60% of the radioactivity had been washed out from the brain by 60 min after injection. The uptake of ^99m^Tc-MAMA-BF (**28**) peaked 30 min after the injection at 1.23%ID/g, and the washout from the brain was slower than that of  ^99m^Tc-BAT-BF (**27**) throughout the time course, which is unsuitable for imaging *in vivo*. The combination of good affinity for A*β* plaques, uptake, and clearance makes  ^99m^Tc-BAT-BF (**27**) a promising probe for the detection of A*β* plaques in the brain. The results of the present study should provide useful information for the development of ^99m^Tc-labeled probes for the imaging of A*β* plaques in the brain, although there are some difficulties associated with the large size of  ^99m^Tc complex in the molecular design of  ^99m^Tc-labeled A*β* imaging probes to enhance the penetration of blood-brain barrier.

## 4. Conclusion

Many PET probes targeting A*β* plaques in the brain have been tested clinically and demonstrated potential utility. Unfortunately, the short half-life (^11^C; 20 min, ^18^F; 110 min) of ^11^C- or ^18^F-labeled probes except ^18^F-FDG limits their use at major academic PET facilities with on-site cyclotrons and sophisticated radiochemistry laboratories. On the other hand, many more hospitals have the capacity to perform SPECT. A*β* imaging probes labeled with SPECT isotopes especially the inexpensive and readily available ^99m^Tc will have more widespread clinical applicability especially in developing countries that cannot afford expensive cyclotron and PET scanners. The development of novel ^123^I- or ^99m^Tc-labeled A*β* imaging probes may lead to simple and convenient SPECT imaging methods for detecting and eventually quantifying A*β* plaques in living brain tissue.

## Figures and Tables

**Table 1:** Inhibition constants (*K*_*i*_, nM)^a^ of compounds for the binding of ligands to aggregates of A*β*(1–40) and A*β*(1–42).

Compound	A*β*(1–40)	A*β*(1–42)
**12**	22.6 ± 3.4	30.0 ± 3.4
**13**	13.2 ± 0.2	15.6 ± 2.4
**14**	29.0 ± 3.2	38.3 ± 8.1
**15**	72.5 ± 8.2	77.2 ± 9.2
Thioflavin T	>1000	>1000
Congo Red	>1000	>1000

^a^Values are the mean ± standard error of the mean for 6 independent experiments.

**Table 2:** Biodistribution of radioactivity after intravenous injection of [^125^I]**12**, [^125^I]**13**, [^125^I]**14**, and [^125^I] **15** in normal mice^a^_._

Tissue	Time after injection (min)			
	2	10	30	60
	[^125^I]**12**			

Blood	1.89 (0.28)	1.39 (0.10)	1.34 (0.07)	1.50 (0.09)
Liver	16.28 (0.90)	25.28 (0.31)	18.61 (1.81)	15.14 (0.89)
Kidney	8.13 (1.28)	5.21 (0.44)	3.85 (0.33)	3.05 (0.25)
Intestine	3.10 (0.61)	7.91 (1.05)	12.84 (1.18)	21.48 (3.17)
Spleen	2.57 (1.54)	2.31 (0.01)	1.76 (0.23)	1.52 (0.29)
Heart	4.87 (0.66)	2.66 (0.12)	1.67 (0.14)	1.28 (0.12)
Stomach^b^	0.78 (0.02)	0.87 (0.22)	1.44 (0.69)	1.80 (0.84)
Brain	4.12 (0.15)	3.68 (0.18)	1.84 (0.12)	1.19 (0.04)

	[^125^I]**13**			

Blood	1.87 (0.18)	1.07 (0.08)	1.20 (0.15)	1.15 (0.16)
Liver	15.41 (0.98)	21.85 (2.14)	15.71 (0.96)	12.40 (2.38)
Kidney	8.33 (1.47)	4.31 (0.28)	3.40 (0.31)	2.32 (0.45)
Intestine	2.24 (0.24)	6.56 (0.83)	12.97 (1.15)	18.64 (2.05)
Spleen	2.72 (0.20)	1.92 (0.33)	1.58 (0.31)	1.18 (0.17)
Heart	5.63 (0.80)	2.47 (0.14)	1.69 (0.06)	1.07 (0.17)
Stomach^b^	0.73 (0.17)	0.63 (0.16)	1.17 (0.40)	1.06 (0.27)
Brain	3.22 (0.15)	3.61 (0.60)	1.89 (0.21)	0.99 (0.10)

	[^125^I]**14**			

Blood	1.87 (0.21)	1.19 (0.17)	0.40 (0.01)	0.23 (0.09)
Liver	8.96 (1.48)	9.01 (0.97)	3.75 (0.47)	1.88 (0.61)
Kidney	7.99 (1.08)	6.30 (1.02)	4.51 (1.59)	1.46 (1.12)
Intestine	3.52 (0.29)	14.39 (0.80)	22.51 (1.11)	30.05 (3.61)
Spleen	2.70 (0.08)	1.38 (0.37)	0.55 (0.30)	3.67 (5.89)
Heart	4.98 (0.41)	2.25 (0.40)	0.84 (0.14)	0.47 (0.22)
Stomach^b^	0.68 (0.06)	0.45 (0.18)	0.55 (0.33)	0.31 (0.07)
Brain	4.00 (0.18)	2.36 (0.33)	0.51 (0.07)	0.17 (0.05)

	[^125^I]**15**			

Blood	2.77 (0.43)	1.58 (0.18)	0.66 (0.03)	0.20 (0.02)
Liver	9.77 (1.89)	8.24 (0.50)	6.80 (0.86)	4.78 (1.09)
Kidney	14.79 (2.59)	15.11 (2.00)	6.45 (0.84)	1.66 (0.62)
Intestine	3.12 (0.37)	11.26 (0.63)	22.01 (1.34)	27.28 (0.48)
Spleen	3.92 (1.18)	1.55 (0.15)	0.56 (0.13)	0.17 (0.06)
Heart	5.51 (0.71)	1.60 (0.18)	0.53 (0.04)	0.12 (0.02)
Stomach^b^	0.89 (0.09)	0.59 (0.16)	1.56 (0.50)	0.81 (0.36)
Brain	3.31 (0.32)	1.90 (0.07)	0.52 (0.03)	0.08 (0.02)

^a^Expressed as % injected dose per gram. Each value represents the mean ± S.D. for 3−5 animals at each interval. ^b^Expressed as % injected dose per organ.

**Table 3:** Biodistribution of radioactivity after injection of ^99m^Tc-labeled benzofuran derivatives in normal mice^a^.

Organ		Time after injection (min)		
	2	10	30	60
^99m^Tc-BAT-BF (**27**)				

Blood	4.40 (0.27)	1.96 (0.06)	1.93 (0.26)	2.15 (0.91)
Liver	21.94 (5.94)	20.87 (1.28)	19.65 (1.31)	15.09 (3.83)
Kidney	10.28 (1.76)	7.90 (0.40)	4.27 (0.18)	2.70 (0.57)
Intestine^b^	1.45 (0.18)	3.68 (0.52)	7.42 (1.62)	9.02 (1.93)
Spleen	5.20 (1.01)	3.09 (0.23)	1.69 (0.21)	1.16 (0.14)
Lung	26.70 (2.27)	6.48 (1.33)	3.51 (0.64)	2.36 (0.48)
Stomach^b^	1.33 (0.57)	1.90 (0.43)	4.09 (1.37)	4.17 (1.92)
Pancreas	4.14 (0.77)	4.57 (0.24)	2.98 (0.38)	1.42 (0.15)
Heart	17.60 (2.60)	8.29 (0.97)	3.28 (1.35)	1.51 (0.25)
Brain	1.34 (0.12)	1.37 (0.18)	0.94 (0.20)	0.56 (0.07)

^99m^Tc-MAMA-BF (**28**)				

Blood	4.13 (0.42)	1.78 (0.25)	2.15 (0.12)	2.24 (0.24)
Liver	20.17 (3.81)	21.62 (2.62)	23.32 (1.59)	20.16 (2.13)
Kidney	7.37 (1.06)	8.09 (1.16)	5.11 (0.29)	3.28 (0.45)
Intestine^b^	0.95 (0.22)	2.13 (0.19)	4.75 (0.93)	5.73 (0.66)
Spleen	4.48 (0.56)	3.69 (0.34)	3.49 (0.61)	2.59 (0.65)
Lung	24.04 (5.17)	7.59 (2.13)	4.24 (0.35)	3.54 (1.26)
Stomach^b^	0.73 (0.21)	2.35 (0.58)	4.94 (0.57)	2.81 (0.51)
Pancreas	2.70 (0.47)	4.00 (1.28)	5.48 (0.61)	3.76 (0.36)
Heart	12.28 (2.20)	10.48 (1.79)	5.05 (0.90)	2.16 (0.34)
Brain	0.74 (0.15)	0.99 (0.22)	1.23 (0.09)	0.89 (0.08)

^a^Each value represents the mean (SD) for 5 mice. Expressed as % injected dose per gram. ^b^Expressed as % injected dose per organ.
